# Tissue-specific deletion of FKBP51 reveals distinct roles in stress responsivity and metabolic regulation

**DOI:** 10.1016/j.molmet.2026.102455

**Published:** 2026-10-08

**Authors:** Margherita Springer, Lea M. Brix, Veronika Kovarova, Sowmya Narayan, Daniela Harbich, Benjamin Haworth, Fatima Feyzanur Mete, Daniel Suñer-Rubio, Sara Stanciu, London Aman, Minnah Irfan, Benjamin Jurek, Shiladitya Mitra, Lotte van Doeselaar, Huanqing Yang, Alessandra Bianco, Maria Clara Sokn, Nils C. Gassen, Samantha Hughes, Mathias V. Schmidt

**Affiliations:** 1 Research Group Neurobiology of Stress Resilience, Max Planck Institute of Psychiatry, 80807 Munich, Germany; 2 International Max Planck Research School for Translational Psychiatry (IMPRS-TP), Munich, Germany; 3 Graduate School of Systemic Neurosciences, Ludwig Maximilians University (GSN-LMU), Munich, Germany; 4 Neurohomeostasis Research Group, Department of Psychiatry and Psychotherapy, University Hospital Bonn, Bonn, Germany; 5 Amsterdam Institute for Life and Environment, Environmental Health and Toxicology, Vrije Universiteit Amsterdam, Amsterdam, the Netherlands

## Abstract

The co-chaperone FKBP51 serves as a critical molecular intersection between environmental stress and metabolic health, yet the specific tissue-level mechanisms driving its systemic effects remain poorly understood. While global FKBP51 deficiency provides robust protection against diet-induced obesity and insulin resistance, this study aimed to isolate the contributions of the brain, skeletal muscle, and adipose tissue using targeted knockout mouse models, cellular assays, and *Caenorhabditis elegans*. In cellular models, both pharmacological antagonism and genetic deletion of FKBP51 enhanced glucose uptake, and silencing its ortholog in *C. elegans* led to increased lipid accumulation, supporting a conserved role in energy metabolism. Neural-specific FKBP51 deletion in mice successfully blunted corticosterone secretion during acute stress, confirming its role in HPA axis regulation, but it did not alter body weight or glucose metabolism under either standard or high-fat diets. Similarly, the deletion of FKBP51 exclusively in adipocytes or muscle failed to impact weight regulation, body composition, or systemic insulin sensitivity. Even the simultaneous deletion of FKBP51 in both adipose and muscle tissues resulted in only minor, sex-specific metabolic shifts, failing to recapitulate the strong phenotype of full-body knockouts. These results indicate that the metabolic protection associated with FKBP51 loss is likely an emergent property of synchronized multi-organ signaling rather than the action of a single dominant tissue. Consequently, effective therapeutic targeting of the FKBP51-stress-metabolism axis may require the concurrent modulation of both central neuroendocrine pathways and peripheral metabolic systems.

## Highlights

• Cellular models and *C. elegans* confirm that FKBP51 plays a conserved role in regulating glucose uptake and lipid accumulation.
• Neural-specific deletion of FKBP51 successfully blunts corticosterone secretion during acute stress.
• Deleting FKBP51 exclusively in either adipose tissue or skeletal muscle fails to impact weight regulation and metabolism.
• Metabolic protection seen in full-body FKBP5 knockouts is an emergent property of synchronized signaling across multiple organs.
• Effective therapeutic targeting of FKBP51 likely requires the concurrent modulation of both central and peripheral systems.

## 1 Introduction

One in three adults are living with overweight or obesity, which is a risk factor for developing chronic illness such as cardiovascular disease, cancer, and metabolic diseases like type 2 diabetes (T2D) [1]. T2D is a complex metabolic disorder characterized by chronic hyperglycemia, insulin resistance, and impaired insulin secretion. Beyond the well-established contributions of a high-caloric diet and sedentary lifestyle, T2D is increasingly recognized as a stress-sensitive disorder, where chronic environmental and psychosocial stressors act as systemic catalysts for metabolic dysfunction [2]. While lifestyle and pharmacological interventions exist [3], the heterogeneity of patient responses suggests that complex molecular regulators - particularly those integrating environmental stress with metabolic control - remain to be fully understood.

*FKBP5*, encoding the co-chaperone FKBP51, has emerged as a critical player in the regulation of stress reactivity and metabolic homeostasis. Originally identified for its role in modulating glucocorticoid receptor sensitivity and the stress response [[4], [5], [6]], FKBP51 has more recently emerged as a pivotal scaffold protein in metabolic homeostasis [7]. Human genetic studies have identified *FKBP5* variants that correlate not only with psychiatric phenotypes [8] but also with T2D-traits and insulin resistance in both adults and children [[9], [10], [11]], as well as decreased responsiveness to exercise interventions [12] and bariatric surgery [13]. Furthermore, early life trauma and chronic stress have been linked to metabolic syndrome through epigenetic modifications, specifically increased DNA methylation of *FKBP5* [14,15]. These clinical observations position FKBP51 as a molecular mediator that may link chronic stress, including stress arising from chronic overnutrition, to metabolic decline [16].

Recent preclinical studies have indeed reinforced this hypothesis and demonstrated that FKBP51 influences energy balance and glucose metabolism. Full body FKBP51 knockout (FKBP51 KO) mice exhibit a remarkable protection from high-fat diet-induced weight gain, improved glucose tolerance, and enhanced insulin signaling in skeletal muscle [17,18]. Moreover, chronic treatment with SAFit2, a novel FKBP51 antagonist, recapitulated these beneficial metabolic effects, underscoring the therapeutic potential of FKBP51 inhibition in metabolic diseases. Mechanistically, FKBP51 has been shown to interact with Akt substrate 160 kDa (AS160), a key substrate of the protein kinase AKT2 involved in glucose uptake, leading to increased phosphorylation of AS160, elevated Glucose transporter 4 (GLUT4) translocation to the plasma membrane, and augmented glucose uptake in skeletal muscle cells. This skeletal muscle-specific regulation may be further modulated by long non-coding RNAs, such as LncFKBP5, which has been identified as a dual regulator of myogenesis and glucose handling [19].

The role of FKBP51 in adipose tissue and the liver is equally compelling. In humans, *FKBP5* expression in adipose tissue increased following glucocorticoid exposure and is strongly associated with insulin resistance and adipocyte dysfunction [9,10]. FKBP51 has been shown to undergo dynamic mitochondrial–nuclear redistribution during adipocyte differentiation [20] and to act as a regulator of adipogenesis [21]. Indeed, CRISPR/Cas9-mediated deletion of *FKBP5* in human preadipocytes significantly altered their metabolic profile [22]. Furthermore, recent evidence suggests that FKBP51 contributes to high fat diet (HFD)-induced metabolic dysfunction-associated steatotic liver disease, further highlighting its systemic reach [23]. Centrally, hypothalamic *Fkbp5* is induced by fasting, and its manipulation in this region directly impacts metabolic control, suggesting a critical role in central energy sensing [24,25]. Previous studies have further demonstrated region- and cell-type-specific functions of FKBP51 within the brain [[25], [26], [27]]. However, it remains unclear to what extent the systemic metabolic effects of FKBP51 are driven by its broader central versus peripheral actions.

While these findings highlight the systemic importance of FKBP51 in metabolic regulation, the specific contributions of FKBP51 in different tissues remain to be elucidated. Given the distinct metabolic functions of skeletal muscle, adipose tissue, and the brain, the sites of highest FKBP51 expression [28], a comprehensive understanding of tissue-specific roles is essential for developing targeted interventions. In this study, we aimed to distinguish the broader central and peripheral contributions of FKBP51 to glucose and energy homeostasis by employing a neural FKBP51 knockout model alongside tissue-specific knockout models targeting skeletal muscle and adipose tissue. Rather than resolving the contribution of individual neuronal populations or brain regions, the neural model was used to investigate the broader contribution of central FKBP51 signaling. Additionally, we used *Caenorhabditis elegans* to explore the evolutionary conserved roles of FKBP51. Our objective is to assess the impact of tissue-specific deletion of FKBP51 on body weight, body composition, and systemic glucose homeostasis, providing a precise blueprint for its potential as a therapeutic target for T2D and related metabolic disorders.

## 2 Materials and methods

### 2.1 Cell culture

C2C12 cells (Cat. # 91,031,101, Sigma Aldrich) were grown at sub-confluency in culture media containing DMEM (Cat. # 11,320,033, Gibco), 10% fetal bovine serum (FBS) (Cat. # 10,500,064, Gibco) and 1% Penicillin-Streptomycin (Cat. # 15,140,122, Gibco). At 90% confluency, differentiation was induced with differentiation media containing DMEM, 2% horse serum (Cat. # 11,591,831, Cytiva) and 1% Penicillin-Streptomycin. FKBP51 knockout (KO) and wild-type (WT) HeLa cell lines, kindly provided by Dr. Nils Gassen (University Clinic Bonn), were generated as described in Martinelli et al. (2024) [29]. Both HeLa cell lines were maintained in DMEM supplemented with 10% FBS and 1% Penicillin-Streptomycin. All cell lines were cultured at 37 °C with 5% CO_2_.

### 2.2 2-Deoxyglucose (2-DG) uptake assay

C2C12 cells (5000 cells/well) were seeded into 96-well clear-bottom plates (Cat. # 732–2605, Thermo Fisher Scientific) with daily media changes. At 90% confluency, the culture media was replaced with differentiation media and refreshed daily. Six days post-differentiation, the cells were serum starved overnight in serum free differentiation media with either vehicle (0.1% DMSO) or 0.6 μM SAFit2, a selective antagonist for FKBP51 [30] which was kindly provided by Prof. Dr. Felix Hausch (Technical University of Darmstadt).

To measure 2-DG uptake, a bioluminescent assay (Cat. #J1341, Glucose Uptake-Glo Assay, Promega) was used according to the manufacturer's recommendation. The assay measures 2-deoxyglucose-6-phosphate (2DG6P), a metabolite of 2-DG that accumulates in cells. The detection reagent included in the kit oxidizes 2DG6P, generating a luminescent signal proportional to the concentration of 2DG6P [31]. One hour before the assay, the cells were glucose starved by replacing the media with glucose-free DMEM (Cat. # 11,966,025, Gibco) with or without 100 nM insulin (Cat. #I5500, Sigma Aldrich). After 1 h, the glucose free media was removed and replaced with phosphate buffered saline (Cat. # 10,010,023, Gibco) containing 0.1 mM 2-DG for 30 min. During each medium change insulin and SAFit2 were included in the corresponding conditions. After 2-DG incubation the cells were lysed with an acidic buffer, neutralized and incubated for 1 h at 25 °C with the 2DG6P detection reagent. The luminescence was measured using a plate reader (Cytation3, Agilent BioTek Instrumentation) with 1 s integration time.

For the HeLa cell experiments, WT and FKBP51 KO cell lines were used without insulin or SAFit2 included in the assay. In this experiment, 7500 cells/well were seeded and the 2-DG uptake assay was performed two days after seeding following the protocol described above.

### 2.3 Preparation of cell lysates and western blotting

Protein was extracted from the HeLa cells lines using RIPA buffer (Cat. # 8990, Thermo Scientific) with protease & phosphatase inhibitors (Cat. # A32961, Thermo Scientific), snap frozen at −80 °C, scraped on ice, sonicated and centrifuged (10,000 rcf, 4 °C for 10 min). Protein concentration of the supernatant was measured using the DC Protein Assay kit according to the manufacturer's instructions (Cat # 5,000,116, Bio-Rad) with bovine serum albumin (BSA) (Cat. # 0163.4, Roth) as a standard. The supernatants were denatured in Laemmli buffer (Cat. # 1,610,737, Bio-Rad) at 95 °C for 5 min and then stored at −20 °C. Cell lysates (20 μg) were separated on a 4–12% Bis-Tris gel (Cat. # NW04127, Invitrogen) and transferred to a nitrocellulose membrane (Cat. # 1,704,158, Bio-Rad) using the Trans-Blot Turbo Transfer System. The membrane was incubated overnight in 5% BSA in Tris buffered saline (Cat. # 1,706,435, Bio-Rad) containing 0.05% Tween-20 (Cat. # 1,706,531, Bio-Rad) (TBS-T) with antibodies targeting FKBP51 (Cat. # A301-430 A, Bethyl) and vinculin (Cat # 13,901, Cell Signaling) at 4 °C. The dilutions used for anti-FKBP51 and anti-vinculin were 1:1000 and 1:500, respectively. The next day, after washing the membrane was incubated with the secondary antibody diluted at 1:10,000 (Cat. # JIM-711-035-152, Jackson ImmunoResearch) in TBS-T containing 5% skim milk (Cat. #T145, Roth). Prior to imaging the membrane was incubated with enhanced chemiluminescence substrate (Cat. # 34,075, Thermo Scientific) and imaged with the ChemiDoc System (Bio-Rad).

Brain, skeletal muscle, and gonadal white adipose tissue were harvested from adult female mice for analysis of FKBP51 expression by Western blot. Protein lysates were prepared with dedicated protocols depending on the tissue. Brain and skeletal muscle tissue were pulverized with a hammer while still frozen, Pulverized tissue (20–30 mg) was weighed into a microcentrifuge tube, combined with T-PER Tissue Protein Extraction Reagent (Cat. # 78,510, Thermo Scientific) at a 1:30 (mg tissue/μL buffer) ratio, supplemented with protease inhibitor (Cat. # 05,892,791,001, Roche) and phosphatase inhibitor cocktails (Cat. # 04,906,837,001, Roche), and homogenized with a Dounce homogenizer. Homogenates were incubated for 1 h at 4 °C under rotation and centrifuged at 14,000×*g* for 30 min at 4 °C to pellet debris. Adipose tissue (110–160 mg) was homogenized in supplemented T-PER buffer (1:5, mg tissue/μL buffer) using a TissueLyser LT (Qiagen) with one stainless steel bead per tube (5 cycles of 1 min shaking at 25 Hz, with 1 min pauses between cycles), then incubated at 4 °C under rotation for 2 h. The liquid phase was transferred to a fresh tube to separate it from the metal bead, then centrifuged at 20,000×*g* for 15 min at 4 °C to separate the pelleted debris and top lipid layer from the protein-containing aqueous phase. This clearing step was repeated three additional times to minimize residual lipid contamination in the final lysate. Total protein concentration was determined using a BCA protein assay (Cat. # 23,225, Thermo Scientific). Samples were normalized to equal protein concentration by dilution in water and Laemmli loading buffer supplemented with 100 mM DTT and heated at 95 °C for 5 min. Equal amounts of protein were resolved by SDS-PAGE and transferred onto nitrocellulose membranes, which were then stained for total protein with Ponceau. Membranes were blocked in TBS-T containing 5% skim milk for 1 h at room temperature, then incubated with FKBP51 primary rabbit antibody (Cat. # 12,210, Cell Signaling) diluted 1:1000 in TBS/0.05% Tween-20 overnight at 4 °C. The following day, blots were washed and probed with Anti-rabbit IgG, HRP-linked Antibody (Cat. # 7074, Cell Signaling) diluted 1:10,000 in TBS-T for 1 h at room temperature. Immuno-reactive bands were visualized using ECL detection reagent (Cat. # 1,705,061, Bio-Rad) on a ChemiDoc Imaging System (Bio-Rad).

### 2.4 C. elegans husbandry and intracellular lipid accumulation assay

The strain used in this study was N2 (wild type) and maintained according to standard protocols [32]. Nematodes were age synchronised using sodium hypochlorate [33]. Synchronized L1 worms were grown at 20 °C for 48 h on RNAi nematode growth media plates [34] containing Nile Red at final concentration of 0.3 μM (Cat. #7726.1 Roth) and seeded with *fkb-6* or the empty vector control. At the L4 stage, worms were selected for microscopy. The lipid accumulation assay was performed according to van de Klashorst et al. (2020) [35]. For microscopy, individual worms were mounted on 1% agarose pads in 50 μM sodium azide (Cat. #08591, Sigma–Aldrich). Fluorescence from the Nile Red labeled lipid droplets in the intestine directly behind the grinder of the second pharyngeal bulb was imaged for each worm. Imaging was carried out with a Leica DMi8 with a DFC900GT camera. Pictures were taken with a 20*x* objective lens the Texas Red filter and LasX v3.9 software (Leica). Image analysis was conducted in ImageJ (https://imagej.net/ij/) with version v1.53, by converting each image to an RGB stack, drawing a 400 × 150 pixels box just behind the pharynx and measuring the fluorescence intensity in arbitrary units within the box.

### 2.5 Mouse models and housing conditions

All experiments and protocols were performed in accordance with the European Communities’ Council Directive 2010/63/EU and were approved by the committee for the Care and Use of Laboratory animals

Of the Government of Upper Bavaria (license numbers: 02-19-10 and 02-23-109). Mice were group housed throughout the experiments and all animals were held in individually ventilated cages (30 cm × 16 cm x 16 cm; 501 cm^2^) serviced by a central airflow system (IVC Green Line GM500, Tecniplast). Animals had ad libitum access to water and food, which contained 14% kcal from fat, 27% from protein, and 59% from carbohydrate (Cat. # 1318, Altromin). The mice were maintained under constant environmental conditions (12:12 h light/dark cycle, 23 ± 2 °C and humidity of 55%). All cages included nesting material and a wooden tunnel for environmental enrichment. All mouse lines in this study (Fkbp51^F/F^, Fkbp51^F/F^ Nes-Cre^+^, Fkbp51^F/F^ Adipo-Cre-ER^T2^, Fkbp51^F/F^ HSA-Cre^+^ and Fkbp51^F/F^ Adipo-Cre-ER^T2^ HSA-Cre^+^) were bred in-house on a C57BL/6 N background and genotyped by the Genetically Engineered Mouse Models Core Unit of the Max Planck Institute of Psychiatry. All experiments were conducted on a pure C57BL/6 N background, on which the Fkbp5 floxed line was established. C57BL/6 N mice carry an intact Nnt allele and reliably develop diet-induced obesity and peripheral insulin resistance, providing a physiologically intact baseline for metabolic phenotyping while avoiding the spontaneous Nnt mutation present in C57BL/6 J mice [36,37]. At the onset of experiments, Fkbp51^F/F^ Nes-Cre^+^ and Fkbp51^F/F^ HSA-Cre^+^ were 7–8 weeks old, Fkbp51^F/F^ Adipo-Cre-ER^T2^ mice were 10 weeks old, and Fkbp51^F/F^ Adipo-Cre-ER^T2^ HSA-Cre^+^ were 9 weeks old.

The Fkbp51^F/F^ Nes-Cre^+^ mouse line was generated by crossing Fkbp51 floxed (Fkbp51^F/F^) [described in Brix et al. (2022) [38] with Nes-Cre^+^ mice, which express Cre recombinase under the nestin promoter to specifically delete Fkbp51 in neurons and glial precursor cells [39]. Similarly, the Fkbp51^F/F^ Adipo-Cre-ER^T2^ and Fkbp51^F/F^ HSA-Cre ^+^ lines where created by crossing Fkbp51^F/F^ mice with Adipo-Cre-ER^T2^ or HSA-Cre^+^ mice, targeting Fkbp51 deletion in adipose tissue [40] and striated muscle [41], respectively, via a tamoxifen inducible Cre (Adipo-Cre-ER^T2^) or Cre expression under the control of the human alpha-skeletal actin promoter (HSA-Cre^+^). For experiments with the Fkbp51^F/F^ Nes-Cre^+^, Fkbp51^F/F^ Adipo-Cre-ER^T2^, Fkbp51^F/F^ HSA-Cre^+^ lines, Fkbp51^F/F^ littermates were used as controls. To generate the double knockout line Fkbp51^F/F^ Adipo-Cre-ER^T2^ HSA-Cre^+^ line (dKO) Fkbp51^F/F^ Adipo-Cre-ER^T2^ females were crossed with Fkbp51^F/F^ HSA-Cre ^+^ males. Males positive for both Cre alleles were bred with Fkbp51^F/F^ Adipo-Cre-ER^T2^ females to generate the experimental cohort. Vehicle treated Fkbp51^F/F^ and Fkbp51^F/F^ Adipo-Cre-ER^T2^ served as littermate controls.

After phenotyping on chow diet, mice were fed a high-fat diet containing 58% kcal from fat, 16.5% kcal from protein and 25.5% kcal from carbohydrates (Cat. #D12331, Research Diets) for the remainder of the experiment. The different conditional knockout lines were studied as independent experimental cohorts at different stages of the project and therefore differed in the age at which metabolic phenotyping and HFD exposure were initiated. Within each experimental cohort, knockout mice and their respective controls were studied at the same age, and no direct statistical comparisons were performed between the different knockout lines.

### 2.6 Tamoxifen induction

To induce Cre recombination in male mice, Fkbp51^F/F^ Adipo-Cre-ER^T2^ and their control littermates were fed a diet containing tamoxifen (LASCRdiet™ CreActive TAM400, LASvendi) for two weeks starting at 10 weeks old and maintained on the diet for two weeks. Female Fkbp51^F/F^ Adipo-Cre-ER^T2^ mice and Adipo-Cre-ER^T2^ HSA-Cre^+^ mice were administered tamoxifen via intraperitoneal (i.p.) injection at a dose of 100 mg/kg body weight for five consecutive days at seven weeks old. Tamoxifen (Cat. #T5648, Sigma Aldrich) was dissolved in 5% v/v ethanol (Cat. # 1.00990, Sigma Aldrich) and 95% v/v corn oil (Cat. #C8267, Sigma Aldrich), mixed overnight at 37 °C protected from light. Littermate controls were injected for five consecutive days with vehicle. Phenotyping started 3–8 weeks following tamoxifen treatment.

### 2.7 Acute restraint stress test

The acute restraint stress test was used to assess the stress response in 28 week-old Fkbp51^F/F^ Nes-Cre^+^ mice and littermate controls as described by Brix et al. (2022) [38]. One hour after the onset of the light phase, each animal was placed in a restrainer with ventilation (50 ml Falcon tube with a hole in the cap and tip to provide room for oxygen and tail movement) for 15 min in their home cage. After 15 min in the restrainer a blood sample was collected by tail cut and then the mouse was released from the restrainer into the home cage. A subsequent blood sample was collected 60 min post stress by tail cut.

### 2.8 Corticosterone measurement

The blood samples from mice during the acute restraint stress paradigm were collected in 1.5 ml EDTA-coated microcentrifuge tubes (Cat. # 078,035, Kabe Labortechnik), and kept on ice until centrifuged at 4 °C, 8000 rpm for 15 min. The plasma was collected into 0.5 ml tubes (Cat # 0030,108,116, Eppendorf) and frozen at −80 °C until measurement. Corticosterone was measured by ELISA (Cat. # RE52211, IBL International) according to the manufacturer's specifications at the Max Planck Institute for Psychiatry's Biomaterial Processing and Repository Unit.

### 2.9 Body composition

We assessed body composition using quantitative Nuclear Magnetic Resonance (qNMR) (Minispec, Bruker) during the animal facility's light period in non-anesthetized, fed mice on a chow diet or a HFD. Mice were briefly placed in restraint tubes, and body composition was determined using a single scan according to the manufacturer's instructions.

### 2.10 Glucose tolerance test (GTT) and insulin tolerance test (ITT)

GTTs were performed after an overnight (14-hour) fast. A first blood sample was collected by tail poke to measure fasting blood glucose, then mice were dosed with 2 g/kg body weight glucose (Cat. #G68270, Sigma Aldrich) dissolved in sterile 0.9% saline (Cat. # 1,636,349, Fresenius Kabi) via i. p. Injection. Glucose was administered intraperitoneally rather than orally in order to assess systemic glucose disposal while minimizing variability arising from gastrointestinal absorption, gastric emptying, and gut-derived incretin responses. Thus, the ipGTT was used as a standardized measure of post-absorptive glucose handling in these experiments. Blood was sampled from the tail poke 15, 30, 60, 90 and 120 min post glucose dosing with a handheld glucose monitor (Cat. # 84,157,377, Contour XT) and test strips (Cat. # 08,884,487, Contour next).

The insulin tolerance test was conducted during the light period after 4 h of fasting. Blood was collected via tail poke prior to insulin dosing, to assess fasting blood glucose. Insulin (Actrapid Penfill, Novo Nordisk) was diluted in sterile saline and administered by intraperitoneal injection with a standardized insulin load of 1 IU/kg body weight. Blood glucose was measured at 15, 30 and 60, 90 and 120 min post insulin injection using a blood glucose monitor and glucose test strips.

For the experiments with the Fkbp5^F/F^ Nes-Cre + line, the GTT was performed at 30 weeks of age and the ITT at 32 weeks of age. HFD feeding began at 35 weeks of age. The GTT and ITT were repeated at 41 and 43 weeks of age, respectively.

For males of the Fkbp5^F/F^ Adipo-Cre-ER^T2^ line, the GTT was performed at 20 weeks old and the ITT was performed at 24 weeks old. HFD feeding began at 25 weeks of age. The GTT and ITT were repeated at 33 and 38 weeks old. For females of the Fkbp5^F/F^ Adipo-Cre-ER^T2^ line, the GTT was performed at 20 weeks old and the ITT at 22 weeks old. HFD feeding began at 24 weeks of age. The GTT and ITT were repeated at 32 and 34 weeks old.

For the Fkbp5^F/F^ HSA-Cre^+^ line, the GTT was performed at 10 weeks old and the ITT at 12 weeks old. After the ITT, HFD feeding began. The GTT was reassessed at 18 weeks old and the ITT at 20 weeks old.

For the Fkbp5^F/F^ Adipo-Cre-ER^T2^ HSA-Cre^+^, the GTT was performed at 12 and 14 weeks old. HFD feeding began at 16 weeks of age. The GTT and ITT were reassessed at 26 and 28 weeks old.

### 2.11 Statistics

Data are presented as mean ± standard deviation (SD) unless otherwise stated, and sample sizes are indicated in the figure legends. Statistical analyses were performed using GraphPad Prism version 10.4.0 (Dotmatics, Boston, MA, USA). For comparisons between two groups, normality was assessed using the Shapiro–Wilk test and equality of variances using an F-test. Normally distributed data were analyzed using an unpaired Student's t-test (t) when variances were equal or Welch's t-tests when variances were unequal. For non-normally distributed data, the Mann–Whitney U test (U) was used. Unless otherwise specified, statistical tests were two-tailed; one-tailed tests (#) were used where a directional hypothesis was specified a priori. Longitudinal and time-course data, including body weight measurements, GTT and ITT were analyzed using two-way repeated-measures ANOVA with time and genotype as factors. When datasets contained missing values, a two-way mixed-effects model (REML) was used instead. Post hoc multiple comparisons were performed using Sidak's correction where appropriate. For GTT and ITT experiments, the area under the curve (AUC) was calculated to assess the overall response and analyzed using the appropriate two-group comparison as described above, based on normality and equality of variances. Male and female mice were analyzed separately. A p value < 0.05 was considered statistically significant. Statistical significance is indicated as ⁎p < 0.05, ⁎⁎p < 0.01, ⁎⁎⁎p < 0.001, and ⁎⁎⁎⁎p < 0.0001. Statistical trends were defined as 0.05 ≤ p ≤ 0.10 and are reported as exact p values.

## 3 Results

### 3.1 Conserved function of FKBP51 in energy metabolism

To explore the role of FKBP51 in energy metabolism, we first investigated the effect on glucose uptake in two cellular models. In C2C12 cells treated with the selective FKBP51 antagonist SAFit2 in the basal and insulin stimulated state, two-way ANOVA revealed a significant effect of SAFit2 (F_(1, 18)_ = 35.35, p < 0.0001) and insulin (F_(1,18)_ = 8.21, p < 0.01) on glucose uptake with no significant interaction between SAFit2 and insulin (F_(1,18)_ = 0.05, p > 0.05). Post-hoc analysis revealed a significant increase in glucose uptake in both basal (p < 0.01) and insulin-stimulated (p < 0.01) conditions (Figure 1A). These findings confirm a previous report of enhanced glucose uptake in primary muscle cells isolated from FKBP51 KO mice [38]. To validate these observations, we conducted experiments with the HeLa cell line where we observed that knockout of FKBP51 significantly increased glucose uptake (t (18) = 6.61, p < 0.0001) further confirming the role of FKBP51 in glucose metabolism (Fig. 1B). Next, we investigated a *C. elegans* model to determine if FKBP51's role in metabolism extends across species. When we silenced *fkb-6*, the closest human ortholog of *FKBP5* in *C. elegans*, we observed a significant increase in lipid droplets (U = 256, p < 0.0001) in the early part of intestine, directly posterior to the grinder of the second pharyngeal bulb. Lipid droplets are the primary fat storage depot of worms which do not contain adipose tissue (Fig. 1C). Together, these observations suggest a consistent and conserved function of FKBP51 in energy metabolism across diverse cellular models and organisms.

**Figure 1:**
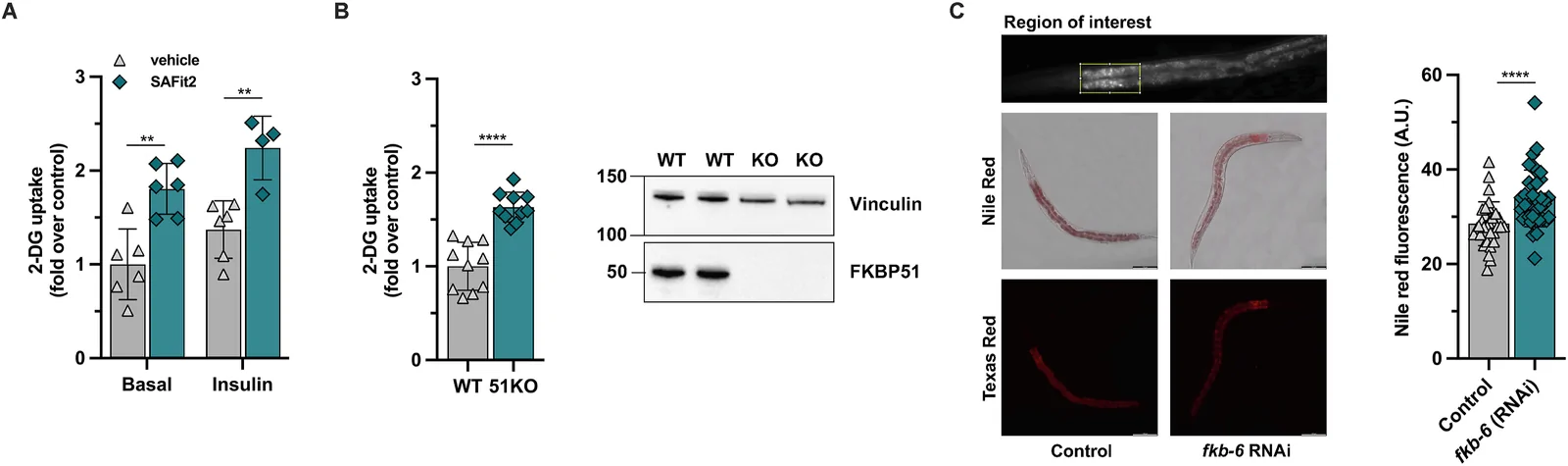
**SAFit2 and *FKBP5* Knockout Increases Glucose Uptake in Cells, While Knockdown Promotes Lipid Accumulation in C. elegans**. (A) SAFit2 Enhances Glucose Uptake in C2C12 Myotubes. C2C12 myotubes were treated with vehicle (0.1% DMSO) or SAFit2 (0.6 μM) in the absence or presence of insulin (0.1 μM). 2-Deoxyglucose (2-DG) uptake was measured and is shown as fold change relative to basal vehicle-treated cells. N = 6 per condition, except SAFit2 + insulin (n = 4). (B) *FKBP5* knockout (51KO) enhances glucose uptake in HeLa cells. Western blot confirms *FKBP5* knockout in HeLa cells with no detectable FKBP51 protein in 51KO cells, while WT cells express FKBP51 (n = 2 per condition). 51KO cells exhibited a significant increase in 2-DG uptake compared to WT controls. 2-Deoxyglucose (2-DG) uptake is presented as fold change relative to WT controls. N = 10 per condition. (C) *Fkb-6* knockdown increases lipid accumulation in *C. elegans*. In ImageJ, a 400 × 150 pixel region of interest (ROI) encompassing the grinder was drawn to quantify fluorescence from Nile Red staining, used here as a proxy for lipid accumulation. Fluorescence images of worms were acquired with a focus on the grinder region of the pharynx using a Texas Red filter. Representative images from control and *fkb-6* (RNAi)-treated worms are shown. Fkb-6 knockdown resulted in visibly enhanced fluorescence in the ROI, indicating increased lipid content. For the control worms (n = 33) and for *fkb-6* RNAi treated worms (n = 37). Data are presented as mean ± SD with individual data points overlaid.

### 3.2 Neuronal FKBP51 deletion affects the HPA axis but not metabolism

Building on previous research investigating the metabolic role of FKBP51 in full body FKBP51 KO mice, we next explored the tissue specific contributions of FKBP51 on metabolism. We hypothesised that the expression pattern may influence distinct yet interconnected metabolic processes. To untangle these complex interactions, we generated tissue specific FKBP51 KO models, targeting deletion in the brain, adipose tissue, and skeletal muscle. Additionally, as adipose tissue and skeletal muscle are the two tissues that abundantly express FKBP51 in the body, we generated an additional double knockout model to simulate a peripheral knockout model and disentangle central vs peripheral effects of FKBP51 deletion on metabolism.

To investigate the role of FKBP51 in the brain, *Fkbp5*^F/F^ mice were bred with Nes-Cre^+^ to generate a KO of FKBP51 in neural cells. Western blot analysis showed a marked reduction in FKBP51 protein levels in brain tissue from KO compared with WT mice (one-tailed Welch's t-test, t (5.021) = 5.710, p < 0.01; Figure 2A). We next assessed stress response, body weight, and glucose metabolism in FKBP51^F/F^ and FKBP51^F/F^ Nes-Cre^+^ in both sexes. The stress response was evaluated by subjecting mice to acute restraint stress and measuring corticosterone levels as an indicator of HPA axis activation. FKBP51^F/F^ Nes-Cre^+^ mice showed significantly lower corticosterone levels than controls in both males (t (23) = 2.45, p <0.05) and females (t (20) = 2.30, p < 0.05; Fig. 2B). This finding is consistent with previous observations in full-body FKBP51 KO mice and further supports a role for brain FKBP51 in regulating HPA axis activity and glucocorticoid signaling during stress [17]. During recovery, corticosterone levels did not differ between genotypes in males (t (20) = 0.35, p > 0.05), whereas females showed a trend toward lower corticosterone levels in FKBP51^F/F^ Nes-Cre^+^ mice (Welch's t-test, t (11.97) = 2.07, p = 0.06; Fig. 2C).

**Figure 2:**
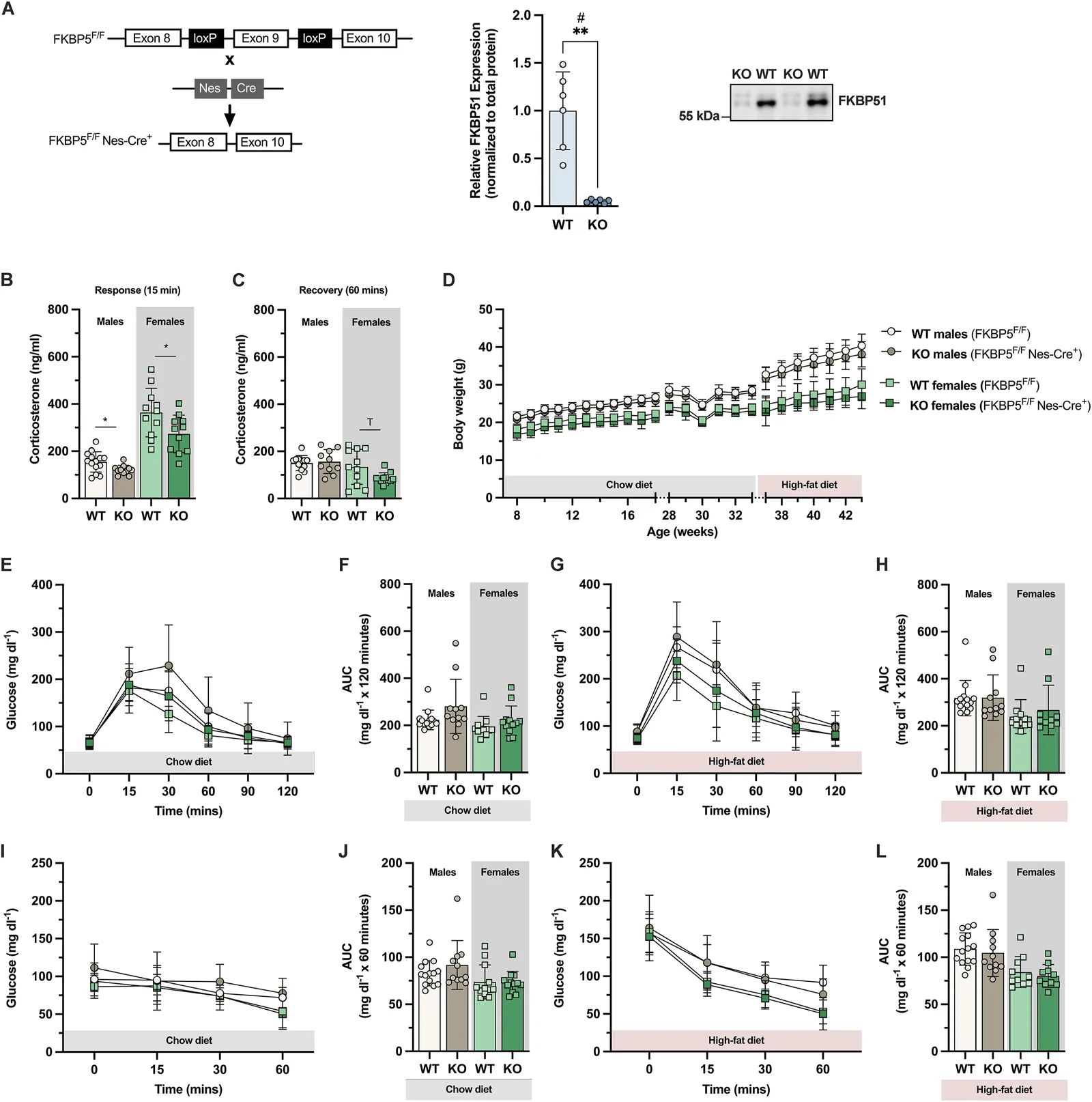
**Brain-Specific FKBP51 Deletion Blunts the Stress Response Without Affecting Body Weight or Glucose Homeostasis**. (A) FKBP5^F/F^ mice were crossed with Nes-Cre^+^ mice to generate mice with FKBP51 deletion in Nestin-expressing cells of the central nervous system. Quantification of FKBP51 protein levels and representative Western blot in brain tissue from FKBP5^F/F^ (WT) and FKBP5F/F Nes-Cre^+^ (KO) mice. For both groups, n = 6. (B) Corticosterone levels in the response phase of the acute restraint stress test. For FKBP5^F/F^ males n = 14 and for all other conditions (FKBP5^F/F^ Nes-Cre^+^ males, FKBP5^F/F^ females and FKBP5^F/F^ Nes-Cre^+^ females) n = 10. (C) Corticosterone levels in the recovery phase of the acute restraint stress. For FKBP5^F/F^ males n = 12 and for FKBP5^F/F^ Nes-Cre^+^ males n = 10. For FKBP5^F/F^ females and FKBP5^F/F^ Nes-Cre^+^ females n = 10. (D) Body weight of mice on a chow and HFD. For FKBP5^F/F^ males n = 14 and for all other conditions (FKBP5^F/F^ Nes-Cre^+^ males, FKBP5^F/F^ females and FKBP5^F/F^ Nes-Cre^+^ females) n = 12. (E) GTT on chow diet. For FKBP5^F/F^ males n = 14 and for FKBP5^F/F^ Nes-Cre^+^ males n = 11. For FKBP5^F/F^ females and FKBP5^F/F^ Nes-Cre^+^ females n = 12 per group (F) Quantification of the GTT shown in D as the AUC (G) GTT after six weeks of HFD feeding. For FKBP5^F/F^ males n = 14 and for FKBP5^F/F^ Nes-Cre^+^ males n = 11. For FKBP5^F/F^ females and FKBP5^F/F^ n = 11 and for FKBP5^F/F^ Nes-Cre^+^ females n = 11. (H) Quantification of the GTT shown in G as the AUC (I) ITT on chow diet. For FKBP5^F/F^ males n = 14 and for FKBP5^F/F^ Nes-Cre^+^ males n = 11. For FKBP5^F/F^ females and FKBP5^F/F^ Nes-Cre^+^ females n = 12 per group. (J) Quantification of the ITT shown in I as the AUC (K) ITT after eight weeks of HFD feeding. For FKBP5^F/F^ males n = 14 and for FKBP5^F/F^ Nes-Cre^+^ males n = 11. For FKBP5^F/F^ females and FKBP5^F/F^ Nes-Cre^+^ females n = 10 per group (L) Quantification of the ITT shown in K as the AUC. For FKBP5^F/F^ males n = 14 and for FKBP5^F/F^ Nes-Cre^+^ males n = 11. For FKBP5^F/F^ females and FKBP5^F/F^ Nes-Cre^+^ females n = 10 per group. Data are presented as mean ± SD.

Body weight was tracked over several weeks in both sexes on chow and HFD. Body weight increased over time in both sexes (p <0.0001). No genotype effect was observed in males (F_(1,24)_ = 2.64, p > 0.05), whereas females showed a significant genotype effect (F_(1,22)_ = 6.01, p = 0.02), although post-hoc comparisons revealed no differences at individual time points. No significant time × genotype interactions were observed in either sex (Fig. 2D).

On chow, blood glucose changed significantly over time during the GTT in both sexes (p < 0.0001), with no genotype effect in males (F_(1,23)_ = 2.93, p > 0.05) or females (F_(1,22)_ = 1.20, p > 0.05). Significant time × genotype interactions were observed in both sexes (males: F_(5,115)_ = 3.24, p < 0.01; females: F_(5,110)_ = 2.85, p < 0.05), although post-hoc comparisons revealed no genotype differences at individual time points (Fig. 2E). AUC also did not differ between genotypes in males (Welch's t-test, t (12.06) = 1.57, p > 0.05) or females (t (22) = 1.20, p > 0.05; Fig. 2F). After six weeks of HFD, glucose similarly changed over time in both sexes (p < 0.0001), with no genotype effect in males (F_(1,23)_ = 0.03, p > 0.05) or females (F_(1,19)_ = 0.62, p > 0.05) and no significant time × genotype interactions (Fig. 2G). AUC was also unaffected by genotype in males (Welch's t-test, t (18.50) = 0.04, p > 0.05) or females (t (19) = 0.71, p > 0.05; Fig. 2H). Together, these findings indicate that central FKBP51 deletion does not markedly affect glucose tolerance under chow or HFD conditions.

Next, insulin sensitivity was assessed by an ITT. On chow, blood glucose decreased significantly over time in both sexes (p < 0.0001), with no genotype effect in males (F_(1,23)_ = 1.12, p > 0.05) or females (F_(1,22)_ = 0.00, p > 0.05) and no significant time × genotype interactions (Fig. 2I). AUC also did not differ between genotypes in males (Welch's t-test, t (14.11) = 1.02, p > 0.05) or females (t (22) = 0.09, p > 0.05; Fig. 2J).

After eight weeks of HFD, blood glucose similarly decreased over time in both sexes (p < 0.0001), with no genotype effect in males (F_(1,23)_ = 0.11, p > 0.05) or females (F_(1,18)_ = 0.38, p > 0.05) and no significant time × genotype interactions (Fig. 2K). AUC was also unaffected by genotype in males (U = 58, p > 0.05) or females (t (18) = 0.62, p > 0.05; Fig. 2L).

### 3.3 FKBP51 deletion in adipose tissue does not impact metabolic control

As central FKBP51 deletion did not affect systemic glucose metabolism, we next examined the role of FKBP51 in peripheral metabolic tissues. Given the high expression of FKBP51 in adipose tissue, we first investigated its role in adipocytes using an inducible adipocyte-specific knockout model generated by crossing Fkbp5^F/F^ mice with Adipo-Cre-ER^T2^ mice. Western blot analysis showed a reduction in FKBP51 protein levels in adipose tissue from KO compared with WT mice (one-tailed Welch's t-test, t (2.135) = 10.40, p < 0.01; Fig. 3A).

**Figure 3:**
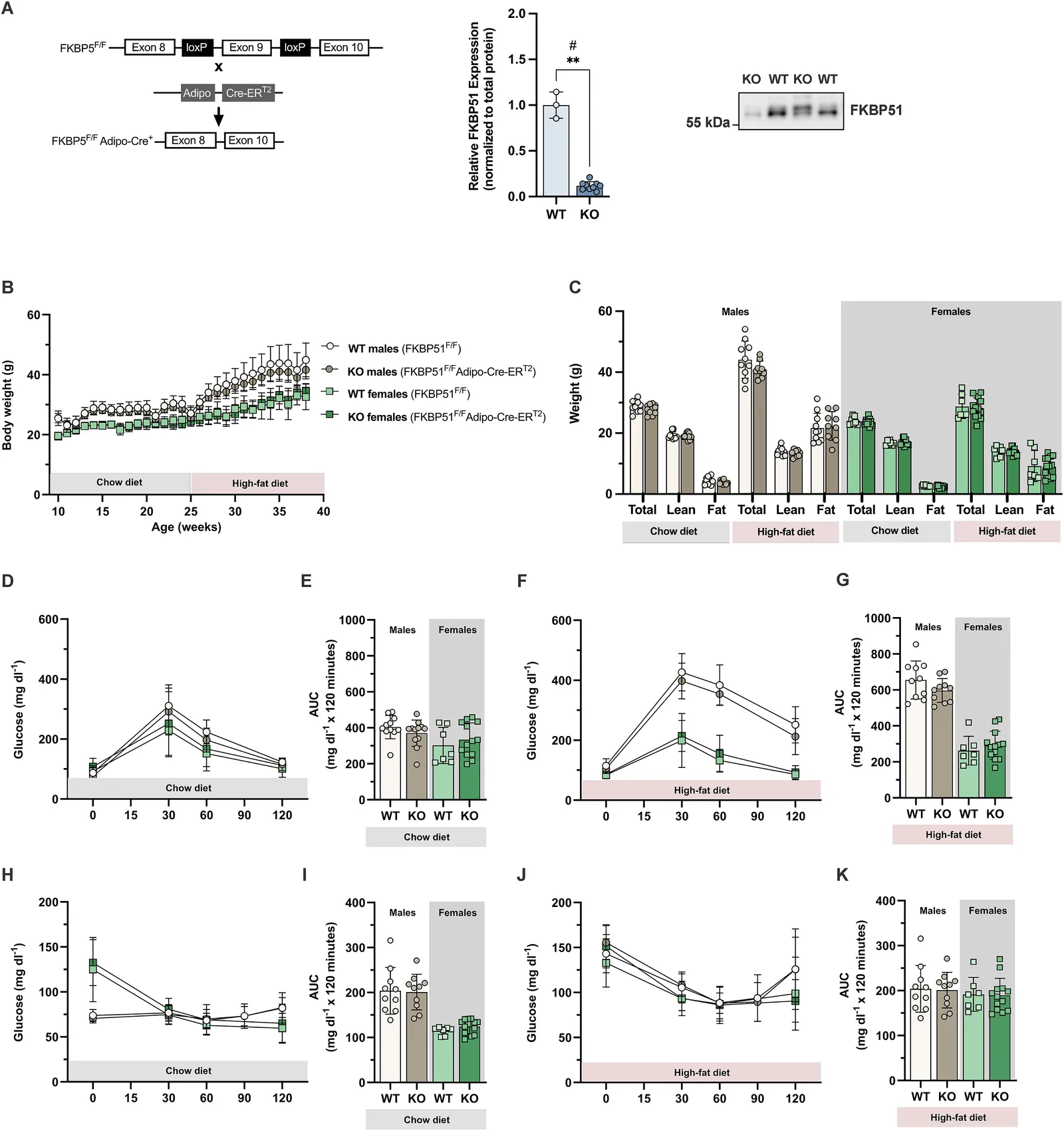
**Adipose-Specific FKBP51 Deletion Does Not Alter Metabolic Phenotypes**. (A) FKBP5^F/F^ mice were crossed with Adipo-Cre-ER^T2^ mice to generate mice with a tamoxifen-inducible deletion of FKBP51 in adipocytes. Quantification of FKBP51 protein levels and representative Western blot in adipose tissue from FKBP5^F/F^ (WT) and FKBP5^F/F^ Adipo-Cre-ER ^T2^ (KO) mice. For WT n = 3; for KO, n = 9. (B) Body weight of mice on a chow and HFD. For males, n = 10 per genotype; for females, FKBP5^F/F^ n = 7 and FKBP5^F/F^ Adipo-Cre-ER^T2^ n = 13. (C) Body composition of mice measured at 19 weeks old while maintained on a chow diet. After 8 weeks on a HFD body composition was measured at 33 weeks old. For male FKBP5^F/F^ mice n = 11 and for male FKBP5^F/F^ Adipo-Cre-ER^T2^ n = 12 for body composition measured during a chow diet. After HFD feeding, FKBP5^F/F^ mice n = 10 and FKBP5^F/F^ Adipo-Cre-ER^T2^ n = 9. For body composition of female mice maintained on a chow diet and HFD; FKBP5^F/F^ n = 7 and for FKBP5^F/F^ Adipo-Cre-ER^T2^ n = 13. (D) GTT on a chow diet with FKBP5^F/F^ male mice n = 11; FKBP5^F/F^ Adipo-Cre-ER^T2^ male mice n = 12; FKBP5^F/F^ female mice n = 7 and FKBP5^F/F^ Adipo-Cre-ER^T2^ female mice n = 13. (E) Quantification of the GTT shown in D as the AUC with FKBP5^F/F^ male mice n = 11; FKBP5^F/F^ Adipo-Cre-ER^T2^male mice n = 12; FKBP5^F/F^ female mice n = 7 and FKBP5^F/F^ Adipo-Cre-ER^T2^ female mice n = 13. (F) GTT after eight weeks of HFD feeding with FKBP5^F/F^ male mice n = 11; FKBP5^F/F^ Adipo-Cre-ER^T2^ male mice n = 10; FKBP5^F/F^ female mice n = 7 and FKBP5^F/F^ Adipo-Cre-ER^T2^female mice n = 13 (G) Quantification of the GTT shown in G as the AUC with FKBP5^F/F^ male mice n = 11; FKBP5^F/F^ Adipo-Cre-ER^T2^ male mice n = 10; FKBP5^F/F^ female mice n = 7 and FKBP5^F/F^ Adipo-Cre-ER^T2^ female mice n = 13 (H) ITT on chow diet at 22 weeks old with FKBP5^F/F^ male mice n = 11; FKBP5^F/F^ Adipo-Cre-ER^T2^male mice n = 12; FKBP5^F/F^ female mice n = 7 and FKBP5^F/F^ Adipo-Cre-ER^T2^ female mice n = 13 (I) Quantification of the ITT on a chow diet shown in H as AUC with FKBP5^F/F^ male mice n = 11; FKBP5^F/F^ Adipo-Cre-ER^T2^ male mice n = 12; FKBP5^F/F^ female mice n = 7 and FKBP5^F/F^ Adipo-Cre-ER^T2^ female mice n = 13 (J) ITT at 34 weeks after 10 weeks on a HFD with FKBP5^F/F^ male mice n = 10; FKBP5^F/F^ Adipo-Cre-ER^T2^ male mice n = 10; FKBP5^F/F^ female mice n = 7 and FKBP5^F/F^ Adipo-Cre-ER^T2^ female mice n = 13 (K) Quantification of the ITT for mice on a HFD shown in panel J as AUC. FKBP5^F/F^ male mice n = 10; FKBP5^F/F^ Adipo-Cre-ER^T2^ male mice n = 10; FKBP5^F/F^ female mice n = 7 and FKBP5^F/F^ Adipo-Cre-ER^T2^ female mice n = 13. Data are presented as mean ± SD.

Following tamoxifen-induced FKBP51 deletion, body weight was monitored in Fkbp5^F/F^ and Fkbp5^F/F^ Adipo-Cre-ER^T2^ mice, which were switched from chow to HFD at 25 weeks. Body weight increased over time in both sexes (p < 0.0001), with no genotype effect in males (F_(1,18)_ = 2.17, p > 0.05) or females (F_(1,18)_ = 0.06, p > 0.05). A significant time × genotype interaction was observed in males (F_(28,504)_ = 1.59, p < 0.05), although post-hoc comparisons revealed no genotype differences at individual time points. No significant interaction was observed in females (Fig. 3B).

Body composition was assessed by qNMR during chow and after HFD feeding. On chow, total body mass, lean mass, and fat mass did not differ between genotypes in males (t (20) = 1.34, t (20) = 0.53, and t (20) = 1.56, respectively; all p > 0.05) or females (t (18) = 0.64, t (18) = 0.46, and t (18) = 1.42, respectively; all p > 0.05). Similarly, after HFD feeding, no genotype differences were observed in total, lean, or fat mass in males (t (17) = 1.43, 1.35, and 0.30, respectively; all p > 0.05) or females (t (18) = 0.30, 0.30, and 0.09, respectively; all p > 0.05; Fig. 3C).

Glucose tolerance was assessed in adipocyte-specific FKBP51 KO mice during chow and HFD feeding. On chow, blood glucose changed significantly over time in both sexes (p < 0.0001), with no genotype effect in males (F_(1,21)_ = 1.14, p > 0.05) or females (F_(1,18)_ = 0.75, p > 0.05) and no significant time × genotype interactions (Fig. 3D). AUC also did not differ between genotypes in males (t (21) = 1.17, p > 0.05) or females (t (18) = 0.72, p > 0.05; Fig. 3E). After HFD feeding, blood glucose similarly changed over time in both sexes (p < 0.0001), with no genotype effect in males (F_(1,18)_ = 2.26, p > 0.05) or females (F_(1,17)_ = 0.07, p > 0.05). No significant time × genotype interaction was observed in males, whereas females showed a significant interaction (F_(3,51)_ = 9.32, p < 0.0001), although post-hoc comparisons revealed no genotype differences at individual time points (Fig. 3F). AUC did not differ between genotypes in males (t (18) = 1.49, p > 0.05) or females (t (18) = 0.77, p > 0.05; Fig. 3G).

Insulin sensitivity was assessed by ITT in adipocyte-specific FKBP51 KO mice during chow and HFD feeding. On chow, blood glucose decreased significantly over time in both sexes (males: p < 0.001; females: p < 0.0001), with no genotype effect in males (F_(1,21)_ = 0.03, p > 0.05) or females (F_(1,18)_ = 2.79, p > 0.05) and no significant time × genotype interactions (Fig. 3H). AUC also did not differ between genotypes in males (t (18) = 0.14, p > 0.05) or females (t (18) = 1.46, p > 0.05; Fig. 3I). After 10 weeks of HFD, blood glucose decreased significantly over time in both sexes (p < 0.0001), with no genotype effect in males (F_(1,18)_ = 0.07, p > 0.05) or females (F_(1,18)_ = 0.0007, p > 0.05) and no significant time × genotype interactions (Fig. 3J). AUC also did not differ between genotypes in males (t (18) = 0.14, p > 0.05) or females (t (18) = 0.11, p > 0.05; Fig. 3K).

Together, these findings indicate that adipocyte-specific deletion of FKBP51 does not significantly affect body weight regulation, body composition, glucose tolerance, or insulin sensitivity under either chow or high-fat diet conditions, suggesting that FKBP51 in adipocytes is not a major driver of systemic metabolic regulation.

### 3.4 FKBP51 deletion in skeletal muscle does not alter metabolic control

We next examined the role of FKBP51 in skeletal muscle, a major metabolic tissue involved in glucose uptake and energy homeostasis. FKPB5^F/F^ mice were crossed with HSA-Cre^+^ mice to generate a skeletal muscle–specific FKBP51 knockout model. FKBP51 protein levels were significantly reduced in skeletal muscle of KO compared with WT mice (one-tailed t-test, t (10) = 5.25, p < 0.001; Figure 4A).

**Figure 4:**
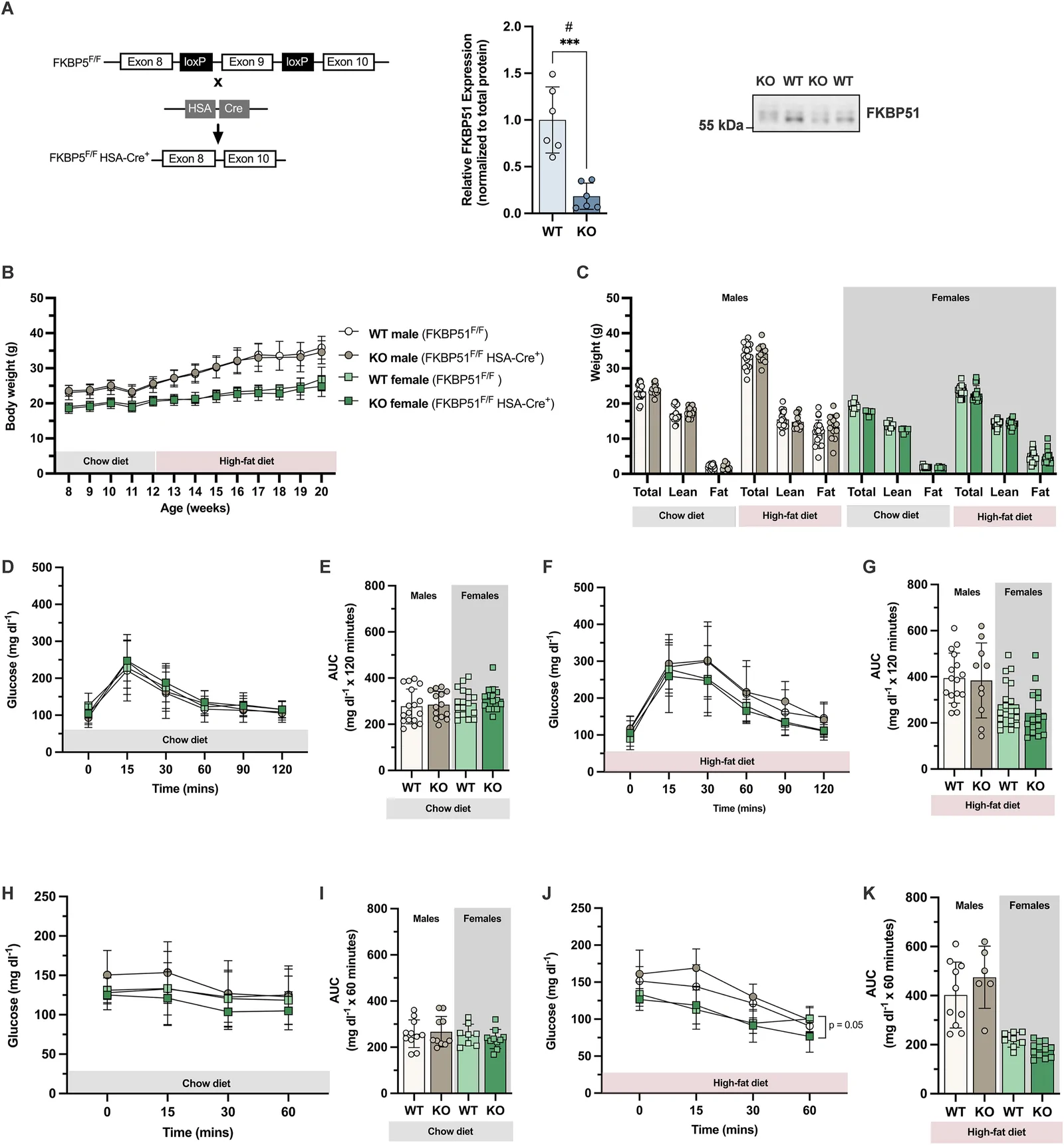
**Muscle-Specific FKBP51 Deletion Has No Effect on Body Weight or Glucose Homeostasis control**. (A) FKBP5^F/F^ mice were crossed with HSA-Cre^+^ mice to generate mice with skeletal muscle–specific deletion of FKBP51. Quantification of FKBP51 protein levels and representative Western blot in skeletal muscle from FKBP5^F/F^ (WT) and FKBP5^F/F^ HSA-Cre^+^ (KO) mice. For both groups, n = 6. (B) Body weight of mice maintained on a chow diet and then switched to a HFD starting at 12 weeks old (C) Body composition on a chow diet was measured at nine weeks old. After six weeks of HFD feeding, body composition was reassessed at 18 weeks old. For FKBP5^F/F^ males n = 19; FKBP5^F/F^ HSA-Cre^+^ n = 14. For females n = 17 per group. (D) GTT on a chow diet at 10 weeks old. For FKBP5^F/F^ males n = 15; FKBP5^F/F^ HSA-Cre^+^ n = 14. For females n = 17 per group (E) Quantification of the GTT shown in D as the AUC (F) The GTT after HFD feeding was reassessed at 19 weeks old. FKBP5^F/F^ males n = 15; FKBP5^F/F^ HSA-Cre^+^ n = 10. For FKBP5^F/F^ females n = 17 and for FKBP5^F/F^ HSA-Cre^+^ n = 15 (G) The GTT after HFD shown in panel F was quantified as AUC (H) The ITT on a chow diet at 12 weeks old FKBP5^F/F^ males n = 11; FKBP5^F/F^ HSA-Cre^+^ n = 10. For FKBP5^F/F^ females n = 8 and for FKBP5^F/F^ HSA-Cre^+^ n = 10 (I) The ITT on a chow diet shown in panel I quantified as the AUC. (J) After HFD feeding the ITT was reassessed at 21 weeks old. FKBP5^F/F^ males n = 10; FKBP5^F/F^ HSA-Cre^+^ n = 6. For FKBP5^F/F^ females n = 12 and for FKBP5^F/F^ HSA-Cre^+^ n = 8 (K) The ITT after HFD feeding shown in panel J as AUC. (G) AUC of the GTT shown in F (H) ITT on chow diet (I) AUC of the ITT shown in H (J) ITT on a HFD (K) AUC of the ITT shown in panel J. Data are presented as mean ± SD.

Body weight was monitored in male and female FKPB5^F/F^ and FKPB5^F/F^ HSA-Cre^+^ mice maintained on chow diet from 7 to 12 weeks of age and subsequently switched to HFD until 20 weeks. Body weight increased over time in both males and females (p < 0.0001), with no overall genotype effect in either sex (males: F_(1,38)_ = 0.036, *p* > 0.05; females: F_(1,32)_ = 1.767, *p* > 0.05). In females, a significant time × genotype interaction was detected (F_(11,342)_ = 1.901, *p* < 0.05); however, post-hoc comparisons revealed no genotype differences at individual time points (Fig. 4B).

Body composition was comparable between genotypes in both sexes under chow and HFD conditions. In males, no genotype differences were detected in total body mass, lean mass, or fat mass on chow (total: t (31) = 0.90; lean: t (31) = 0.94; fat: t (31) = 0.90; all p > 0.05) or HFD (total: t (31) = 0.67; lean: t (31) = 0.11; fat: t (31) = 0.01; all *p* > 0.05). Similarly, females showed no differences on chow (total: t (14) = 2.07; lean: t (14) = 1.94; fat: t (14) = 0.76; all p > 0.05) or HFD (total: t (31) = 1.06; lean: t (31) = 0.21; fat: t (31) = 0.24; all p > 0.05; Fig. 4C).

Glucose tolerance was unaffected by skeletal muscle–specific FKBP51 deletion under either diet. During chow GTT, glucose levels changed over time in both sexes (p < 0.0001), with no genotype effect in males (F_(1,31)_ = 0.25, *p* > 0.05) or females (F_(1,31)_ = 0.10, *p* > 0.05; Fig. 4D). GTT AUC was likewise comparable between genotypes in males (t (31) = 0.35, *p* > 0.05) and females (t (31) = 0.32, p > 0.05; Fig. 4E). Following HFD, glucose levels again changed over time in both sexes (p < 0.0001), with no genotype effect in males (F_(1,34)_ = 0.10, *p* > 0.05) or females (F_(1,13)_ = 0.01, *p* > 0.05; Fig. 4F). GTT AUC remained comparable between genotypes in males (t (31) = 0.28, p > 0.05) and females (t (31) = 0.17, p > 0.05; Fig. 4G).

Under chow conditions, glucose levels changed significantly over time during the ITT in both sexes (p < 0.05), with no effect of genotype in males (F_(1,19)_ = 0.86, p > 0.05) or females (F_(1,16)_ = 1.28, p > 0.05; Fig. 4H). ITT AUC did not differ between genotypes in males (t (19) = 0.59, p > 0.05) or females (t (18) = 0.34, p > 0.05; Fig. 4I). Following HFD, glucose levels changed significantly over time in both sexes (p < 0.0001), with no overall genotype effect in males (F_(1,22)_ = 3.48, p > 0.05) or females (F (_1,18)_ = 1.08, p > 0.05). In females, a significant time × genotype interaction was detected (F_(3,54)_ = 3.02, p < 0.05), with post-hoc analysis identifying a genotype difference at 60 min (p < 0.05; Fig. 4J). Despite this transient difference, ITT AUC was comparable between genotypes in males (t (22) = 1.28, p > 0.05) and females (t (18) = 1.64, p > 0.05; Fig. 4K).

Together, these findings indicate that skeletal muscle–specific FKBP51 deletion has little effect on body composition, glucose tolerance, or insulin sensitivity under chow or HFD conditions, despite a transient difference in the female HFD ITT.

Given the limited metabolic phenotypes following tissue-specific FKBP51 deletion, we investigated FKBP52 expression and downstream metabolic signaling. FKBP52 abundance was unchanged in brain, adipose tissue, and skeletal muscle. In brain, no changes in AS160 signaling were detected. Within the AKT2 pathway, phosphorylated (p) AKT2 and the pAKT2/AKT2 ratio showed trends toward lower levels in FKBP51 KO mice (pAKT2: t (10) = 2.06, p = 0.07; pAKT2/AKT2: t (10) = 2.16, p = 0.06).

In adipose tissue, the pAS160/AS160 ratio showed a trend toward lower levels in FKBP51 KO mice (t (10) = 2.07, p = 0.07). Total AKT2 showed a trend toward increased levels (t (10) = 2.02, p = 0.07), whereas pAKT2 (t (10) = 2.93, p < 0.05) and the pAKT2/AKT2 ratio (t (10) = 4.42, p < 0.01) were significantly reduced.

In skeletal muscle, total AS160 (t (10) = 2.86, p < 0.05) and pAS160 (t (10) = 4.13, p < 0.01) were significantly increased in FKBP51 KO mice. Total AKT2 (Welch's t-test, t (5.671) = 7.46, p < 0.001) and pAKT2 (Welch's t-test, t (6.605) = 4.38, p < 0.01) were also significantly increased (Supplementary Fig. 1).

Together, these findings do not support compensation through altered FKBP52 abundance but indicate tissue-specific adaptations in downstream metabolic signaling following FKBP51 deletion.

### 3.5 Moderate and sex-specific metabolic effects of combined adipocyte and skeletal muscle FKBP51 deletion

We next examined the metabolic effects of simultaneous FKBP51 deletion in adipocytes and skeletal muscle by crossing Fkbp5^F/F^ Adipo-Cre-ER^T2^ mice with Fkbp5^F/F^ HSA-Cre^+^ mice to generate double knockout (dKO) mice (Fig. 5A). Following tamoxifen-induced deletion, body weight increased over time in both sexes (p < 0.0001), with no genotype effect in males (F_(1,9)_ = 0.54, p > 0.05) or females (F_(1,12)_ = 3.36, p > 0.05) and no significant time × genotype interactions (Fig. 5B).

**Figure 5:**
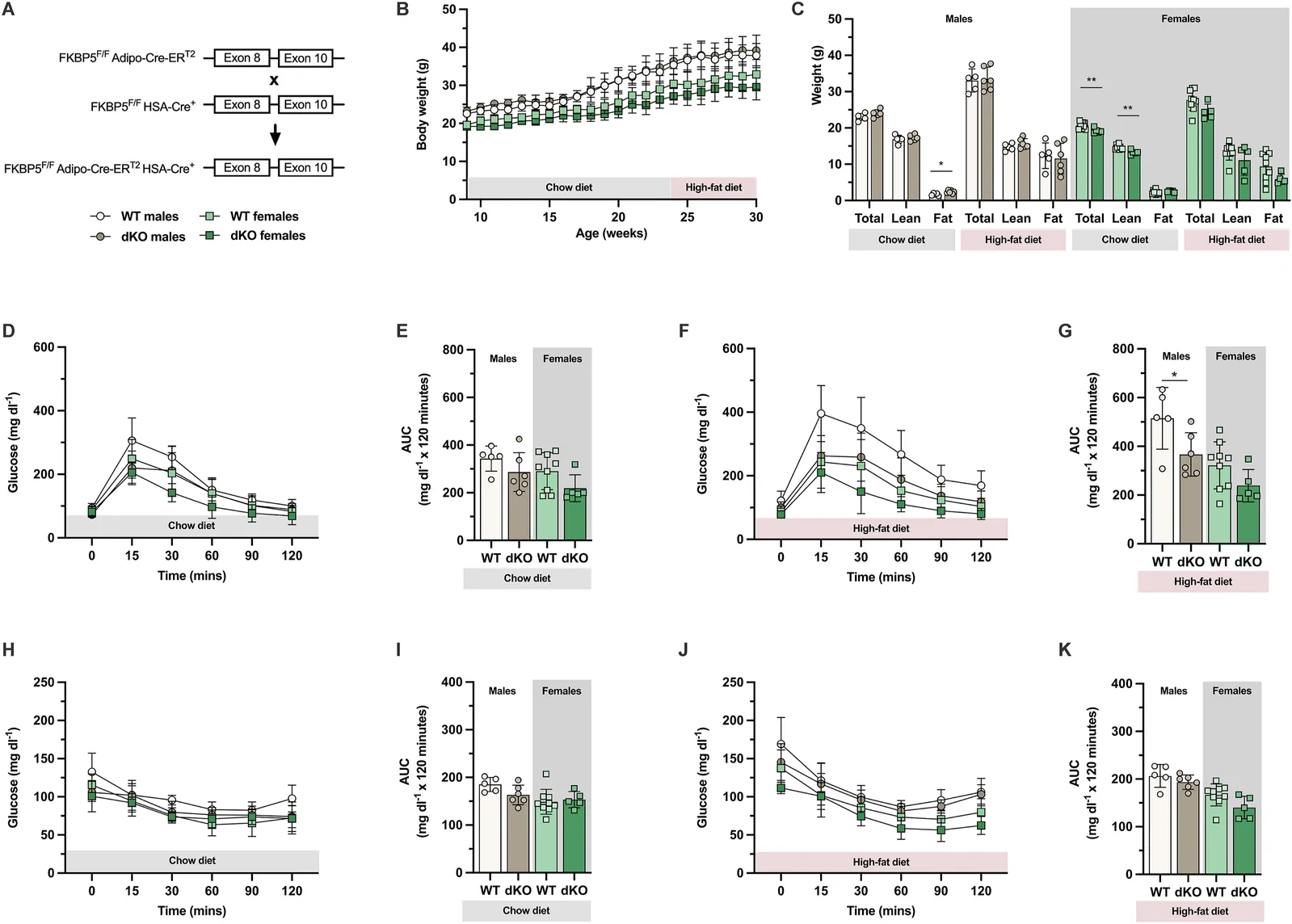
**Adipose- and Muscle-Specific FKBP51 Deletion (dKO) Leaves Metabolism Unchanged, With Subtle Female-Specific Differences**. (A) The dKO line was generated by breeding FKBP5^F/F^ Adipo-Cre-ER^T2^ females with FKBP5^F/F^ HSA-Cre^+^ males. Mice positive for both Cre alleles were dosed with 100 mg/kg tamoxifen for five consecutive days. Vehicle-treated FKBP5^F/F^ and FKBP5^F/F^ Adipo-Cre-ER^T2^ mice served as littermate controls (B) Body weight of mice maintained on a chow and then switched to a HFD at 16 weeks old. (C) Body composition of mice on a chow diet at 10 weeks old and then measured again at 22 weeks old (D) The GTT was evaluated at 12 weeks old while mice were maintained on a chow diet (E) The GTT in panel D quantified as the AUC (F) Glucose tolerance was reassessed after HFD feeding in 26 week old mice (G) The GTT in panel F quantified as AUC (H) The ITT was evaluated at 14 weeks old while mice were maintained on a chow diet (I) The ITT in panel H quantified as the AUC (J) Insulin tolerance was reassessed after HFD feeding in 28 week old mice (K) The ITT shown in panel J quantified as the AUC. For control males n = 5: dKO males n = 6; control females n = 9 and dKO females n = 6. Data are presented as mean ± SD. ⁎p < 0.05, ⁎⁎p < 0.01.

Body composition showed modest sex-specific differences during chow feeding. Male dKO mice had increased fat mass (t (9) = 3.02, p < 0.05), whereas female dKO mice had lower total body mass (t (12) = 3.08, p < 0.01) and lean mass (t (12) = 4.00, p < 0.01). Following HFD feeding, no genotype differences in body composition were observed in either sex (Fig. 5C).

During the GTT on a chow diet, blood glucose changed significantly over time in both sexes (p < 0.0001), with no genotype effect in males (F_(1,9)_ = 2.71, p > 0.05) or females (F_(1,12)_ = 2.99, p > 0.05). Significant time × genotype interactions were observed in males (F_(5,45)_ = 2.60, p < 0.05) and females (F_(5,60)_ = 2.47, p < 0.05). Post-hoc comparisons showed a genotype difference only at baseline in males (p < 0.05), with no differences at individual time points in females (Fig. 5D). GTT AUC did not differ between genotypes in males (t (9) = 1.34, p > 0.05) or females (t (12) = 1.80, p > 0.05; Fig. 5E).

After HFD, blood glucose changed significantly over time in both sexes (p < 0.0001). Male dKO mice showed a significant genotype effect (F**(1,9)** = 5.70, p < 0.05) and time × genotype interaction (F_(5,45)_ = 3.27, p < 0.05), although post-hoc comparisons revealed no genotype differences at individual time points. No genotype effect (F_(1,12)_ = 2.65, p > 0.05) or significant interaction was observed in females (Fig. 5F). GTT AUC was significantly reduced in male dKO mice (t (9) = 2.28, p < 0.05), but did not differ between female genotypes (t (12) = 1.70, p > 0.05; Fig. 5G).

During the chow ITT, blood glucose changed significantly over time in both sexes (p < 0.0001). A significant genotype effect was observed in males (F_(1,9)_ = 6.00, p < 0.05), although post-hoc comparisons revealed no differences at individual time points, whereas no genotype effect was detected in females (F_(1,12)_ = 0.01, p > 0.05). No significant time × genotype interactions were observed (Fig. 5H). ITT AUC did not differ between genotypes in males (t (9) = 1.99, p > 0.05) or females (t (12) = 0.34, p > 0.05; Fig. 5I).

After HFD, blood glucose changed significantly over time in both sexes (p < 0.0001), with no genotype effect in males (F_(1,9)_ = 1.30, p > 0.05) or females (F_(1,12)_ = 4.66, p > 0.05) and no significant time × genotype interactions (Fig. 5J). ITT AUC did not differ between male genotypes (t (9) = 1.10, p > 0.05), but tended to be lower in female dKO mice (t (12) = 2.03, p = 0.07; Fig. 5K). Overall, these results show that combined deletion of FKBP51 in adipocytes and skeletal muscle produces limited effects on body weight, body composition, and glucose and insulin tolerance under chow and HFD conditions.

## 4 Discussion

FKBP51 has emerged as a compelling link between stress biology and metabolic disease. Genetic variation in *FKBP5* is associated with metabolic traits in humans [9,12,13], and previous work in full-body FKBP51 knockout mice and pharmacological inhibition with SAFit2 demonstrated robust protection from diet-induced metabolic dysfunction, including improved glucose tolerance and enhanced insulin signaling in skeletal muscle [17]. However, the tissue-specific contribution of FKBP51 to systemic metabolic regulation has remained unclear. In this study, we combined complementary approaches using cellular systems, an invertebrate model, and multiple tissue-specific knockout mouse lines to dissect how FKBP51 influences glucose and energy homeostasis. Our results support a conserved role of FKBP51-related proteins in cellular glucose handling and lipid storage, although the directionality of these effects appears context and species dependent. Further, our data reveal that tissue-specific deletion of FKBP51 in brain, adipose tissue, or skeletal muscle alone is not sufficient to recapitulate the metabolic phenotype reported in full-body FKBP51 deficiency.

We first confirmed that FKBP51 influences glucose uptake in cellular models. Treatment of C2C12 myotubes with SAFit2 increased glucose uptake both basally and following insulin stimulation, consistent with prior reports showing enhanced glucose disposal in skeletal muscle cells lacking FKBP51 [17]. In parallel, FKBP51 knockout in HeLa cells also increased glucose uptake, providing additional evidence that both pharmacological and genetic manipulation of FKBP51 results in negative regulation of glucose uptake. Although HeLa cells do not represent a metabolically specialized tissue, they provide a simplified cellular model for assessing the cell-autonomous consequences of FKBP51 deletion. Together, these findings support the hypothesis that FKBP51 influences glucose transport and/or intracellular glucose metabolism in a cell-autonomous manner, independent of systemic signals. To explore whether FKBP51's metabolic role is evolutionarily conserved, we also used *C. elegans* as a simplified model of energy storage. Silencing *fkb-6*, the closest ortholog of mammalian *FKBP5* [42], increased lipid droplet accumulation. This observation supports the broader interpretation that FKBP51-related proteins regulate metabolism across species. Importantly, the directionality of the phenotype differs from mammalian full-body FKBP51 knockout mice, which are protected from diet-induced weight gain.

Given FKBP51's established role as a glucocorticoid receptor co-chaperone and its expression in the brain, we next investigated brain-specific FKBP51 knockout mice. Consistent with prior work, deletion of FKBP51 in the brain blunted corticosterone responses to acute restraint stress, confirming that central FKBP51 contributes to HPA axis regulation [43]. This aligns with the broader literature showing that FKBP51 acts as an intracellular feedback regulator of glucocorticoid signaling and shapes stress responsivity, specifically through its action in the brain [[44], [45], [46]]. Despite this clear neuroendocrine phenotype, brain-specific FKBP51 deletion did not significantly affect body weight, glucose tolerance, or insulin sensitivity under either chow or high-fat diet conditions. This result suggests that altering FKBP51-mediated stress signaling in the brain is not sufficient to produce a detectable difference in the metabolic parameters tested here. FKBP51 is expressed in several brain regions implicated in the regulation of energy homeostasis, including hypothalamic nuclei such as the paraventricular nucleus, arcuate nucleus, and ventromedial hypothalamus, as well as the hippocampus and amygdala [47]. Previous studies further indicate that the resistance of global FKBP51 knockout mice to diet-induced weight gain is not primarily attributable to reduced food intake, as cumulative food intake remains largely unchanged. Instead, the metabolic phenotype has been associated with increased energy expenditure and altered glucocorticoid receptor sensitivity [17]. These observations suggest that the metabolic effects of central FKBP51 may depend on region-specific mechanisms rather than a generalized effect on feeding behavior. Accordingly, FKBP51-dependent metabolic regulation in the brain may rely on specific neuronal populations or circuits that cannot be resolved using the broad Nestin-Cre deletion strategy employed here. The broad neural deletion employed here was designed to assess the contribution of central FKBP51 signaling as a whole, rather than to resolve the functions of individual brain regions or neuronal populations. Accordingly, the absence of a pronounced metabolic phenotype in this model should not be interpreted as excluding region- or cell-type-specific metabolic functions of FKBP51. Indeed, prior work has shown that FKBP51 in defined hypothalamic regions can influence metabolic outcomes, suggesting that spatial specificity may be critical for translating altered neuroendocrine signaling into measurable metabolic effects [48,49]. Thus, while the present model is informative for distinguishing broader central from peripheral FKBP51 actions, it cannot determine the contribution of individual neuronal populations to metabolic regulation. To explore the possibility of compensatory adaptations, we assessed FKBP52 expression [50] and downstream AS160 and AKT2 signaling across the three tissue-specific knockout models. Total FKBP52 expression was unchanged in brain, adipose tissue, and skeletal muscle, suggesting that compensation through altered FKBP52 abundance is unlikely. However, tissue-specific changes in downstream signaling were observed. In brain, trends toward reduced pAKT2 and pAKT2/AKT2 were detected, while adipose tissue showed altered AKT2 signaling together with trends in AS160 and AKT2-related measures. In skeletal muscle, total and phosphorylated AS160 and AKT2 were increased, although their respective phosphorylation ratios remained unchanged. Together, these findings suggest that FKBP51 deletion produces distinct tissue-specific adaptations in metabolic signaling, which may contribute to the limited systemic metabolic phenotypes observed in the individual knockout models. However, whether these signaling changes represent compensatory mechanisms or direct consequences of FKBP51 deletion remains to be determined. Finally, it remains possible that metabolic consequences of altered HPA axis reactivity would be more apparent under paradigms that directly combine chronic stress exposure with a metabolic challenge.

We next tested the hypothesis that – rather than in the brain – FKBP51 in peripheral metabolic tissues drives systemic glucose homeostasis. Adipose-specific deletion of FKBP51 did not alter body weight, body composition, glucose tolerance, or insulin sensitivity in either chow- or HFD-fed conditions. Similarly, muscle-specific FKBP51 deletion produced no significant differences in these metabolic readouts. These findings contrast with previous work in full-body FKBP51 knockout mice, where protection from diet-induced metabolic impairment was observed and where improved insulin signaling in muscles was proposed as a key mechanism. Our results suggest that FKBP51's role in skeletal muscle may be more complex than a cell-autonomous regulator of glucose uptake. While FKBP51 inhibition and deletion increase glucose uptake in muscle cells *in vitro*, this effect may be buffered *in vivo* by compensatory pathways. Additionally, muscle glucose uptake is strongly influenced by systemic factors including insulin secretion, hepatic glucose output, adipokine signaling, and glucocorticoid tone [51,52]. Therefore, the lack of a phenotype in adipose-specific FKBP51 knockout mice suggests either that FKBP51 is not a limiting factor in adipocyte metabolic function under the conditions tested, or that the relevant FKBP51-dependent processes occur in non-adipocyte compartments (e.g., immune cells, stromal cells, or sympathetic innervation). Similarly, the *in vivo* absence of a phenotype in muscle-specific knockout mice may reflect the requirement for coordinated FKBP51 modulation across multiple tissues to shift whole-body glucose handling. We tested this hypothesis in a model of combined deletion of FKBP51 in muscle and adipose tissue to determine whether simultaneous loss of FKBP51 across major peripheral metabolic compartments could recapitulate aspects of the metabolic phenotype observed in the full body FKBP51 KO mouse. The selection of skeletal muscle and adipose tissue for the combinatorial knockout was guided by our previous molecular characterization of FKBP51 expression, which identified white adipose tissue and skeletal muscle as major peripheral sites of FKBP51 protein expression [17]. Neural FKBP51 deletion was examined separately to distinguish central neuroendocrine from peripheral metabolic contributions. While combining neural, adipose, and skeletal muscle deletion could further address interactions between these compartments, such an approach would increasingly mirror systemic FKBP51 deficiency rather than dissect the contribution of central and peripheral deletion. The data obtained from double knockout (dKO) mice lacking FKBP51 in both adipose tissue and skeletal muscle had subtle and sex-specific differences. Female dKO mice showed a tendency toward reduced body weight, particularly during high-fat diet feeding, and exhibited lower lean mass on chow. In contrast, male dKO mice showed a transient increase in fat mass on chow, which was no longer present under HFD conditions. Glucose tolerance showed a consistent trend toward impairment in control mice of both sexes, and female mice showed a tendency for reduced insulin sensitivity under HFD. The emergence of subtle phenotypes in the dKO mice - which were absent in the single-tissue lines - suggests a non-linear relationship between FKBP51 expression and metabolic control. It appears that the metabolic protection observed in full-body deficiency is not the sum of independent tissue effects, but rather an emergent property of synchronized FKBP51 loss across connected neuroendocrine and peripheral signalling.

This study has a number of limitations. First, the different mouse lines underwent metabolic phenotyping and HFD exposure at different ages. Although genotype comparisons were performed within age-matched experimental cohorts, differences in age should be considered when qualitatively comparing phenotypic patterns across the different knockout models. Although the present study assessed body weight, body composition, glucose tolerance, and insulin sensitivity, comprehensive metabolic cage phenotyping was not performed. Therefore, subtle genotype-dependent differences in food intake, energy expenditure, substrate utilization, or feeding patterns cannot be excluded. Future studies employing indirect calorimetry and detailed food-intake monitoring under both chow and high-fat diet conditions will be important to determine whether altered energy intake or expenditure contributes to the metabolic effects of FKBP51. A further limitation of the present study is that food intake and energy expenditure were not systematically assessed. As mice were maintained under group-housed conditions, individual food intake could not be quantified using conventional food-weighing approaches. Therefore, subtle genotype-dependent differences in energy intake or expenditure cannot be excluded. Future studies incorporating detailed food-intake monitoring and indirect calorimetry will be important to determine whether altered energy balance contributes to the metabolic effects of FKBP51.

In summary, our study provides a high-resolution dissection of the tissue-specific contributions of FKBP51 to metabolic regulation. While our *in vitro* and *C. elegans* data reinforce the cell-autonomous role of FKBP51 in modulating glucose handling and lipid dynamics, the lack of a robust metabolic phenotype in single-tissue knockout mice suggests a high degree of systemic resilience. The subtle, sex-specific phenotypes emerging in our dual muscle-adipose knockout model indicate that FKBP51 likely governs energy homeostasis through a coordinated, multi-organ network rather than a single dominant tissue. These findings have significant implications for drug development, as they suggest that while systemic FKBP51 antagonism remains a promising therapeutic strategy for T2D, the metabolic benefits may rely on the simultaneous modulation of both central stress–regulatory axes and peripheral metabolic mechanisms. Future research should focus on the synergistic crosstalk between these compartments to fully unlock the therapeutic potential of the FKBP51-stress-metabolism axis.

## CRediT authorship contribution statement

**Margherita Springer:** Formal analysis, Methodology, Validation, Visualization, Writing – original draft. **Lea M. Brix:** Investigation. **Veronika Kovarova:** Investigation. **Sowmya Narayan:** Investigation. **Daniela Harbich:** Investigation. **Benjamin Haworth:** Investigation. **Fatima Feyzanur Mete:** Investigation. **Daniel Suñer-Rubio:** Investigation. **Sara Stanciu:** Investigation. **London Aman:** Investigation. **Minnah Irfan:** Investigation. **Benjamin Jurek:** Investigation. **Shiladitya Mitra:** Investigation. **Lotte van Doeselaar:** Investigation. **Huanqing Yang:** Investigation. **Alessandra Bianco:** Investigation. **Maria Clara Sokn:** Investigation. **Nils C. Gassen:** Investigation, Methodology, Writing – review & editing. **Samantha Hughes:** Investigation. **Mathias V. Schmidt:** Writing – review & editing, Conceptualization, Supervision, Project administration, Funding acquisition.

## Declaration of competing interest

The authors declare that they have no known competing financial interests or personal relationships that could have appeared to influence the work reported in this paper.

## Data Availability

Data will be made available on request.

## Appendix A Supplementary data

Figure S1 FKBP52 expression and downstream AS160 and AKT2 signaling following tissue-specific FKBP51 deletion. Protein levels of FKBP52, AS160, pAS160, pAS160/AS160, AKT2, pAKT2, and pAKT2/AKT2 were quantified in (A–G) brain from FKBP5^F/F^ (WT) and FKBP5^F/F^ Nes-Cre^+^ (KO) mice (n = 6 per genotype), (H–N) adipose tissue from FKBP5^F/F^ (WT) and FKBP5^F/F^ Adipo-Cre-ER^T2^ (KO) mice (WT n = 3; KO n = 9), and (O–U) skeletal muscle from FKBP5^F/F^ (WT) and FKBP5^F/F^ HSA-Cre^+^ (KO) mice (n = 6 per genotype). Proteins are shown in the order listed above and were normalized to total protein and expressed relative to WT controls. Data are presented as mean ± SD with individual data points overlaid. ⁎p < 0.05, ⁎⁎p < 0.01, ⁎⁎⁎p < 0.001.
