# Supplementary material for: Tissue-specific deletion of FKBP51 reveals distinct roles in stress responsivity and metabolic regulation
Source: Mol Metab. 2026 Oct 8;114:102455. doi: 10.1016/j.molmet.2026.102455 (PMC13696148; doi:10.1016/j.molmet.2026.102455)
Supplement: Supplementary file 1 [file mmc1.pdf]

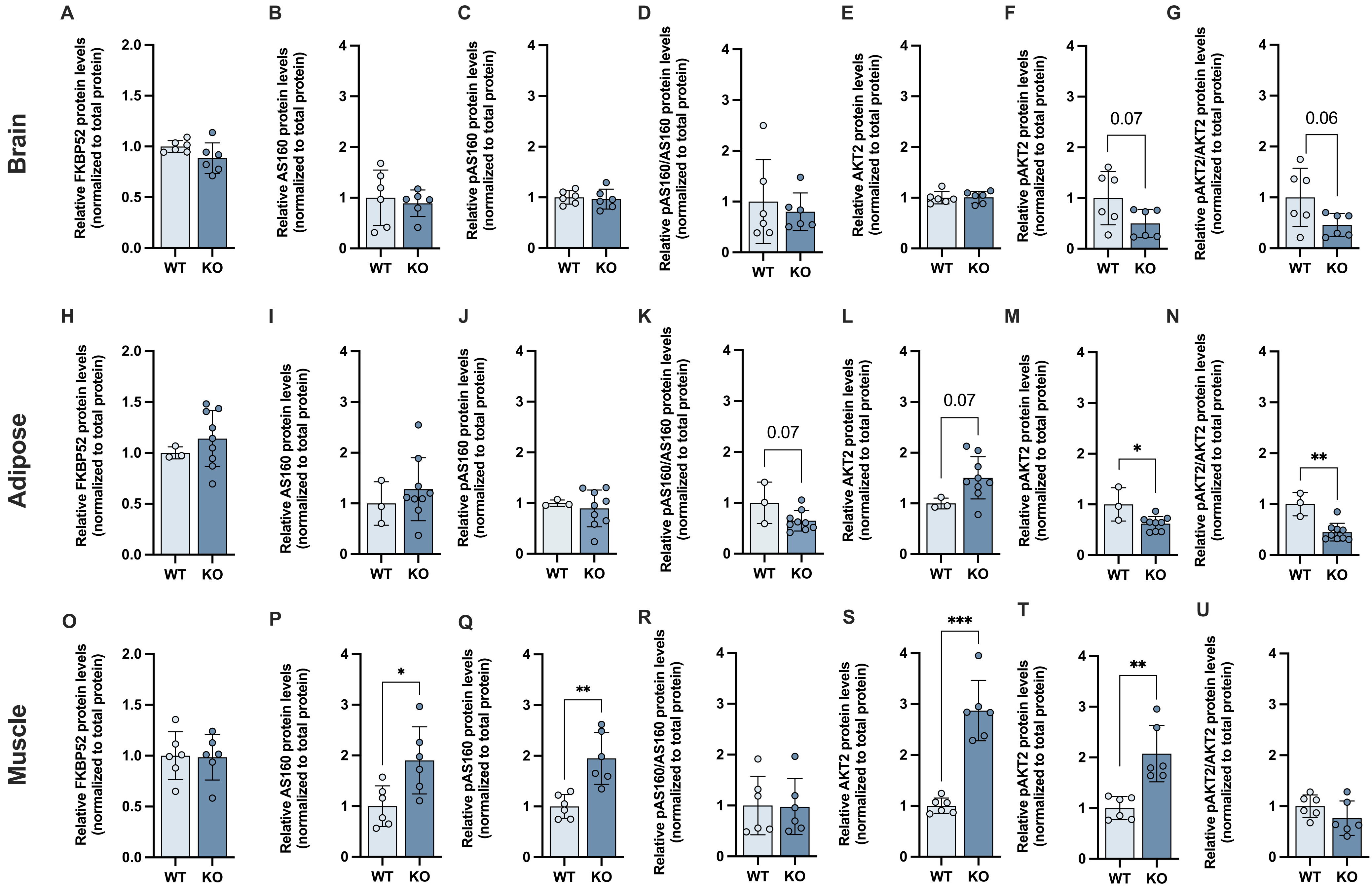

A
B
C
D
E
F
G
Brain
Relative FKBP52 protein levels (normalized to total protein)
Relative AS160 protein levels (normalized to total protein)
Relative pAS160 protein levels (normalized to total protein)
Relative pAS160/AS160 protein levels (normalized to total protein)
Relative AKT2 protein levels (normalized to total protein)
Relative pAKT2 protein levels (normalized to total protein)
Relative pAKT2/AKT2 protein levels (normalized to total protein)
0.07
0.06
H
I
J
K
L
M
N
Adipose
0.07
0.07
*
**
O
P
Q
R
S
T
U
Muscle
*
**
***
**
WT
KO
